# Dynamical criticality during induction of anesthesia in human ECoG recordings

**DOI:** 10.3389/fncir.2014.00020

**Published:** 2014-03-25

**Authors:** Leandro M. Alonso, Alex Proekt, Theodore H. Schwartz, Kane O. Pryor, Guillermo A. Cecchi, Marcelo O. Magnasco

**Affiliations:** ^1^Center for Studies in Physics and Biology, The Rockefeller UniversityNew York, NY, USA; ^2^Department of Anesthesiology, Weill Cornell Medical CollegeNew York, NY, USA; ^3^Laboratory for Neurobiology and Behavior, The Rockefeller UniversityNew York, NY, USA; ^4^Department of Neurological Surgery, Weill Cornell Medical CollegeNew York, NY, USA; ^5^IBM, Thomas J. Watson Research CenterYorktown Heights, NY, USA

**Keywords:** criticality, anesthesia, ECoG, depth of anesthesia monitoring, consiousness, dynamical systems

## Abstract

In this work we analyze electro-corticography (ECoG) recordings in human subjects during induction of anesthesia with propofol. We hypothesize that the decrease in responsiveness that defines the anesthetized state is concomitant with the *stabilization* of neuronal dynamics. To test this hypothesis, we performed a moving vector autoregressive analysis and quantified stability of neuronal dynamics using eigenmode decomposition of the autoregressive matrices, independently fitted to short sliding temporal windows. Consistent with the hypothesis we show that while the subject is awake, many modes of neuronal activity oscillations are found at the edge of instability. As the subject becomes anesthetized, we observe statistically significant increase in the stability of neuronal dynamics, most prominently observed for high frequency oscillations. Stabilization was not observed in phase randomized surrogates constructed to preserve the spectral signatures of each channel of neuronal activity. Thus, stability analysis offers a novel way of quantifying changes in neuronal activity that characterize loss of consciousness induced by general anesthetics.

## 1. Introduction

It has been suggested that neural systems operate in a critical regime similar to phase transitions in physics, given several computational desirable features of such states represented by the statistics of the thermodynamic variables (Chris, 1990). Evidence for *statistical* criticality is based on the observation that various aspects of neuronal activity such as avalanches observed in local field potentials and action potentials in tissue preparations and in animal models (Gireesh and Plenz, 2008; Ribeiro et al., 2010), as well as magneto-encephalography (MEG) and electro-corticography (ECoG) in human subjects (He et al., 2010; Shriki et al., 2013), exhibit long tailed-distributions well approximated by power laws. The critical regime provides important functional benefits; quantities such as dynamic range and information transmission are optimized near criticality (Shew and Plenz, 2013).

More recently, the *dynamical* aspect of criticality has been brought into focus, as a similarly desirable feature not fully captured by steady-state statistics such as avalanche size distributions (Magnasco et al., 2009; Chialvo, 2010; Mora and Bialek, 2011; Beggs and Timme, 2012); a perturbation in an extended dynamical system that is close to a critical point will neither decay nor explode, thus allowing for long range communication across the entire system. In contrast, if the system is far from criticality (therefore stable), perturbations damp out and no information integration takes place beyond the characteristic damping time scale (Tononi, 2008).

While models of self-organized criticality exhibit both dynamically and statistically critical behavior (Bak et al., 1987; Gil and Sornette, 1996), the two aspects of criticality are not necessarily related. The winnerless network provides an illuminating example: under very generic conditions, neural systems can display a phase space determined by heteroclynic orbits connecting saddle nodes (i.e., at least one unstable manifold), such that the resulting dynamics are quasi-periodic cycles over the nodes, without necessarily exhibiting statistically critical distributions (Rabinovich et al., 2001; Aguiar et al., 2011; Ashwin et al., 2011). A model connecting statistical and dynamical criticality in neural systems was proposed recently by Magnasco et al. (2009). They consider an abstract model in which the activity of a set of neurons is encoded in a *N*-dimensional vector $\vec{x}$ which evolves in time according to a *N* × *N* connectivity matrix *A*, characterized by its set of *N* eigenvalues {λ_*n*_}. By assuming anti-Hebbian dynamics for the connectivity matrix a very rich dynamical scenario emerges. The eigenvalues of the matrix *A* evolve toward the dynamically critical point *Re*(λ*n*) ≈ 0 ∀ *n* and the solutions of the model exhibit complex spatio-temporal dynamics, as well as long tailed avalanche distributions and other signatures of statistical criticality. Consistent with this observation, experimental evidence of both statistical and dynamical criticality was reported in human ECoG recordings; however, the precise mechanism by which critical dynamics occur has not been investigated. The analysis showed that the eigenvalues crowd near the critical line, and moreover that task performance (finger tapping) implies a subtle but significant *decrease* in dynamical criticality, presumably because the modes related to motor execution impose higher stability (Solovey et al., 2012). Of note, signatures of statistical criticality were not strongly affected by task performance.

If indeed dynamical criticality is a useful feature of brain activity rather than an epiphenomenon, stability of neuronal dynamics ought to be modulated by the behavioral state of the subject. Here, we hypothesized that a particularly dramatic change in stability accompanies changes in the level of wakefulness (consciousness). When the brain is awake and displaying complex behavior its dynamical state ought to be close to a bifurcation point; marginally stable modes contribute to long range interactions across the system. Conversely when higher-order functions associated with wakefulness have been diminished and eventually completely disrupted by anesthetics, brain dynamics should exhibit more stability. In other words, anesthesia induction should lead to stabilization of brain dynamics.

Changes in the level of arousal (wakefulness) have been historically quantified using spectral analysis of neuronal activity. In this view, decrease in the level of wakefulness is reflected in the increase and prevalence of low frequency oscillations and the concurrent decrease in the high frequency oscillations reviewed in Brown et al. (2010). While this is true for some states of decreased arousal such as slow wave sleep, this association breaks down during other states in which arousal is similarly depressed such as rapid eye movement (REM) sleep for instance. Furthermore, state of general anesthesia can be characterized by different spectral signatures depending on the specific choice of anesthetic agent (Maksimow et al., 2006). This makes current modes of detecting the “depth of anesthesia” unreliable (Avidan et al., 2011).

Lack of clear association between changes in the spectral content of brain signals and level of arousal is not entirely surprising. It is likely that the overall level of wakefulness is a consequence of the interactions among many brain regions rather than any specific feature of neuronal activity observed at any one region taken in isolation. Therefore, more recent efforts have been aimed at detecting decreases in arousal using connectivity measures based on spectral coherence as well as mutual information and phase relationships among brain activity recorded simultaneously at multiple locations (Imas et al., 2005; Cimenser et al., 2011; Lee et al., 2012). While this connectivity analysis does suggest that integration of information between different brain regions may be decreased when the level of wakefulness is reduced, it is not trivial to relate changes in connectivity to the changes in global dynamics of the brain.

To address the dynamics, we fitted vector autoregressive (VAR) models to ECoG signals collected directly from the cortex of human subjects as they were gradually induced into the state of general anesthesia. These models were independently fit to short temporal windows with an arbitrarily large overlap. Thus, while we assume that the dynamics are locally linear and stationary over a short temporal window, on a longer time scale the dynamics are expected to be arbitrarily non-linear and non-stationary. This locally linear approximation allows us to quantify the changes in stability of brain activity in terms of temporal evolution of the distribution of eigenmodes of the fitted models. As previously reported (Solovey et al., 2012), we found a prevalence of critical eigenmodes across the entire recordings. However, the stability of the models shows statistically significant differences across different stages of induction. While the distribution of eigenvalues changes in non-trivial ways, high frequency modes become more damped as anesthesia is induced. Moreover, modes closer to criticality, regardless of frequency, show a gradual shift to stability spanning several drug volleys over approximately 20 min.

This work is organized as follows. In the next section we describe the induction protocol and the analysis method. We present our results in section 3. In section 4 we summarize and discuss our findings.

## 2. Methods

All experimental protocols were approved by the IRB at the Weill Cornell Medical College (protocol number 1106011763). After obtaining informed consent, three subjects undergoing surgical treatment for intractable epilepsy were enrolled in this study. Subdural electrode grids and strips (Ad-tech, Medical Instruments Corp., Racine, WI) were implanted for the purposes of localization of the epileptogenic loci. The location and the number of electrodes were determined by the clinical considerations (temporal lobe for all subjects in this study). After the initial implantation of the subdural electrodes, the subjects underwent video and EEG monitoring, duration of which was dictated solely by clinical considerations (1–2 weeks in these subjects). The recordings analyzed in this work were obtained during induction of anesthesia for the second craniotomy performed after completion of this observation period. During induction of anesthesia (see below), blood pressure, ECG, heart rate, pulse oxymetry, and end tidal carbon dioxide were monitored and maintained within normal limits. Patients were given supplemental oxygen via nasal cannula.

After obtaining baseline recordings (without any pre-medication) anesthesia was gradually induced using target controlled infusions of propofol using pharmacokinetic parameters derived by Schnider et al. (1999), administered using STANPUMP. Target propofol concentration was increased slowly while the level of sedation was accessed using responses to simple verbal commands. Propofol infusion continued until subjects lost the ability to respond to verbal commands. At this point additional propofol, opioids, and neuromuscular blockers were administered (at the discretion of the anesthesia provider) and trachea was intubated. Recordings were terminated at this point.

Recordings were obtained using SynAmps^2^ (Neuroscan) using DC coupled recording. Data were acquired at 10 KHz. 64 channels of ECoG signals were acquired in each subject. While both conventional EEG and ECoG are thought to primarily reflect the sum of synchronized postsynaptic potentials of neurons in the vicinity of the electrode, the invasive nature of the ECoG signals allows for much greater signal to noise ratio and significantly improves spatial and temporal resolution of the signals.

No online filtering was performed. ECoG data was collected from three human subjects as they were induced into general anesthesia. For all subjects, the infusion started 60 s into the recording and the concentration of anesthetics was increased every 300 s. For **Subject 1**, propofol infusion started 60 s into the recording. 360 s into the recording the subject reports being awake. 510 s into the recording the subject no longer responds. 960 s into the recording the subject is given additional drugs and intubated. For **Subject 2**, propofol was incremented at 300, 600, and 900 s. At 660 s the subject opened eyes. 720 s into the recording the subject no longer responded. 1140 s into the recording subject was given additional drugs and was intubated. For **Subject 3**, propofol infusion started 60 s into the recording. The concentration was increased every 300 s and maintained constant before and after. 900 s into the recording the subject no longer responded to verbal commands or light taps on the shoulder. 1200 s into the recording subject was given additional drug and was intubated.

Data was bandpass filtered at 0.1 − 500 Hz and detrended in segments of 10 s. We applied notch filters at 60 ± 2, 120 ± 2, and 180 ± 2 Hz. Finally, the amplitude of the signal in each channel was normalized by its standard deviation. For our analysis data was partitioned in equally sized windows of τ = 200 ms centered every *t*_*j* + 1_ − *t_j_* = Δ = 100 ms. In each window, we assumed that the dynamics is locally linear and fitted a vector auto regressive model (VAR) of order *p* = 1.

(1) $$y_{n+1}=Ay_{n}+\;u_{n}$$

where *y_n_* are the fitted values, *A* ∈ ℝ^*N* × *N*^ is the matrix to be estimated and *u_n_* is assumed to be white noise. Here, *y_n_* ∈ ℝ^*N*^ is a multivariate time series that represents the recorded activity in all channels at time *t_n_* and *A* corresponds to the LAG 1 correlation between channels. A comprehensive treatment of this model and its estimation can be found in Lütkepohl (2006). In this work we used a python implementation of Schnider's et al. algorithm to estimate *A* (Schneider and Arnold, 2001). This procedure yields a set of matrices *A_j_* which govern the stability properties of the VAR model at time *t*_*j*_. In order to address changes in the stability of the fitted models we considered the distribution of the modulus of the eigenvalues at each time step. Also, since our underlying hypothesis corresponds to a continuum model we performed a transformation in order to obtain a correspondence between the eigenvalues of *A*_*j*_ and the timescales of the dynamics. Let λ_*j*_ = *ρ*_*j*_ *e*^*i*ϕ^ be the eigenvalue corresponding to the *j*-th mode, the frequency of the mode is given by

(2) $$f_{j}=\frac{\phi_{j}}{2\pi dt}$$

while the growth rate (timescale) of the mode is given by

(3) $$\tau_{j}=\frac{log(\rho_{j})}{dt}$$

Here *dt* = $\frac{1}{S_{f}}$ = 0.0001 s, where *S*_*f*_ is the sampling frequency of the recordings. A mode with eigenvalue λ is critical if

(4) ‖λ‖ = 1

In practice however, we call a mode critical if ǁλǁ ≈ 1 (thus τ ≈ 0 s). These are modes which are close to alternate their behavior between damping and growth (Strogatz, 2006).

The distributions so obtained were compared to the initial distribution (prior to induction) by means of two statistical tests. Kolmogorov-Smirnov (KS) tests the null hypothesis that the distributions are the same and yields the maximal difference of the cumulative distributions to quantify for the changes. Wilcoxon rank-sum (W) tests the null hypothesis that the distributions are the same against the alternative hypothesis that they are shifted and returns a *z*-value to account for the magnitude of the shift. If the values of the subsequent distributions are smaller than those of the reference distribution (awake state) then *z* > 0, therefore, an increase of the *z*-value indicates an increase of the stability.

We settled on a VAR-1 model because the main results related to the effect of anesthesia are robust for VAR-2 and VAR-3 models. We have explored window sizes ranging from 100 ms to 1 s and found no significant changes. Our method was tested against surrogate data obtained by phase randomization of the signal; for each channel we computed the Fourier transform of the signal, changed the phase value by a random number [drawn from a flat distribution in (0, 2*π*)] and transformed back to obtain the surrogated signals. Note that by construction this procedure preserves the power spectrum of each signal.

## 3. Results

**Figure 1:**
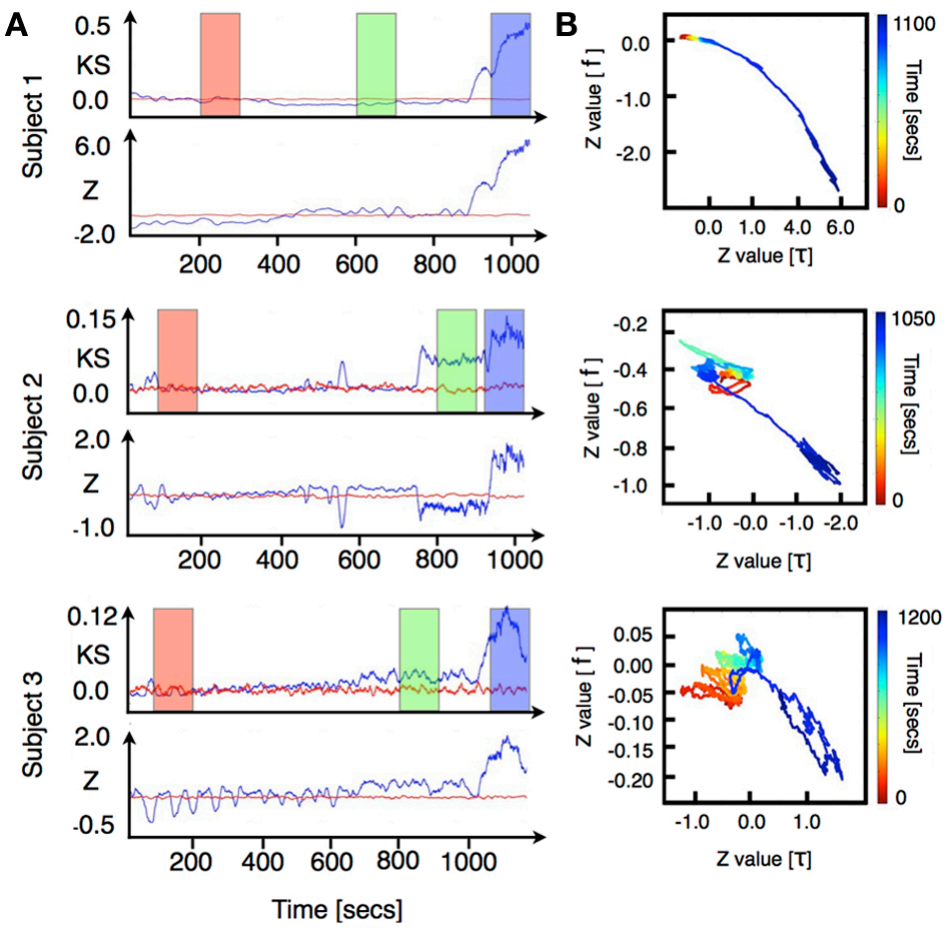
**ECoG signals were recorded from three human subjects as they were induced into general anesthesia**. Data was locally fitted with VAR(1) models in windows of 200 ms every 100 ms (see methods). The linear stability of each model is compared to the awake state by means of two statistical tests. **(A)** top rows: Kolmogorov Smirnov test. For each model we plot the KS statistics of comparing the fitted distribution of eigenvalues against the awake state. **(A)** bottom rows: The distribution of time scales is compared using Wilcoxon test. Both quantities were averaged in time intervals of 10 s. The stability properties of locally fitted VAR(1) models change as the subjects undergo anesthesia. We defined three different segments (color rectangles) which were used for subsequent figures. **(B)** Changes in the frequency and stability of the eigenmodes. We compared the distributions of frequencies and time scales using a Wilcoxon test. In each figure, the vertical axis shows the *z*-value of comparing the frequency distributions whereas the horizontal axis shows the same test for the stability parameters distributions. The color code represents time elapsed since the beginning of the recording. In this representation all realizations yield qualitatively similar results: as the subjects are induced, the fitted frequencies shift to higher values at the same time they become more damped.

We performed VAR analysis on three human subjects as they were induced into general anesthesia. Our primary focus was to detect changes in the distribution of the stability parameters ǁλ_*j*_ǁ during induction of anesthesia. To quantify changes in the stability of the models we used two non-parametric statistical tests [Kolmogorov-Smirnov (KS) and Wilcoxon rank-sums(W)]. The results of this analysis are shown in Figure 1A [for each subject top row shows (KS) and bottom row shows (W)]. To improve visualization the results were smoothed using moving average windows of 10 s. The distribution of eigenmodes computed over different windows during the awake state fluctuates. To scale the observed differences in stability during induction of anesthesia by these spontaneous fluctuations, we computed the time average of both KS and W statistics over the awake period (1 min) and subtracted this value from the curves shown in Figure 1A. In all cases, the temporal average of the *p*-values behaves similarly to the KS-Z values. During the first minutes of the procedure we find that *p* ≈ 0.75, thus, the null hypothesis that the distributions are the same cannot be safely rejected. However, we find a drastic drop of the *p*-value concomitant with changes in KS-Z values. For the regions indicated in blue and green (Figure 1A), the average *p*-value of both tests are in the range of 0.2−0.3 suggesting that the distributions have changed. While the KS test simply indicates that the distributions of stability parameters during awake and anesthetized states are different, the increase in the *z*-values of the Wilcoxon test implies that ǁλ_*j*_ǁ tends to decrease with induction of anesthesia, i.e., the dynamics is becoming more stable. Note that the change in the distribution of the stability parameter is not observed in phase randomized surrogates (red curves in Figure 1A). Thus, the observed changes in stability are not given by the spectral properties of neuronal activity.

**Figure A1:**
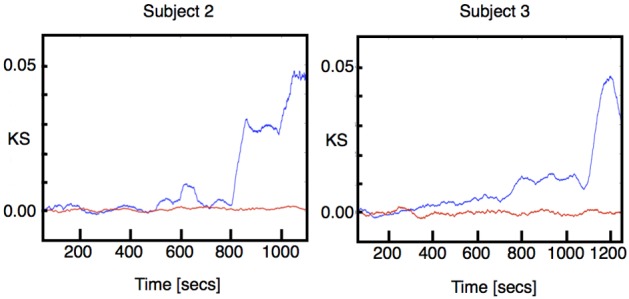
**Model robustness**. The figure shows the same analysis presented in Figure 1A (2), but for a VAR-3 model. The results are almost identical to those for VAR-1.

Note that in general the eigenvalues of the autoregressive matrices fitted to the ECoG signals are complex numbers whose real and imaginary parts give rise to the timescale τ and frequency *f* of the corresponding eigenmode (see Equations 2, 3). While Figure 1A focused just on changes in the distribution of the stability parameters, Figure 1B shows changes in both the distribution of timescales (abscissa) and bulk frequencies (ordinate) treated independently. Time elapsed since the onset of experiment is color coded from red (awake) to blue (anesthetized). The bulk evolution of the eigenmodes is consistent in all subjects: as induction progresses, modes shift to higher frequencies while they become more stable. To validate that the results obtained with the VAR-1 model are robust, we show in Figure A1 (included as Appendix) the same analysis as in Figure 1A implemented with a VAR-3 model. As it can be seen, the changes in the distribution of eigenmodes are almost identical to those for VAR-1.

**Figure 2:**
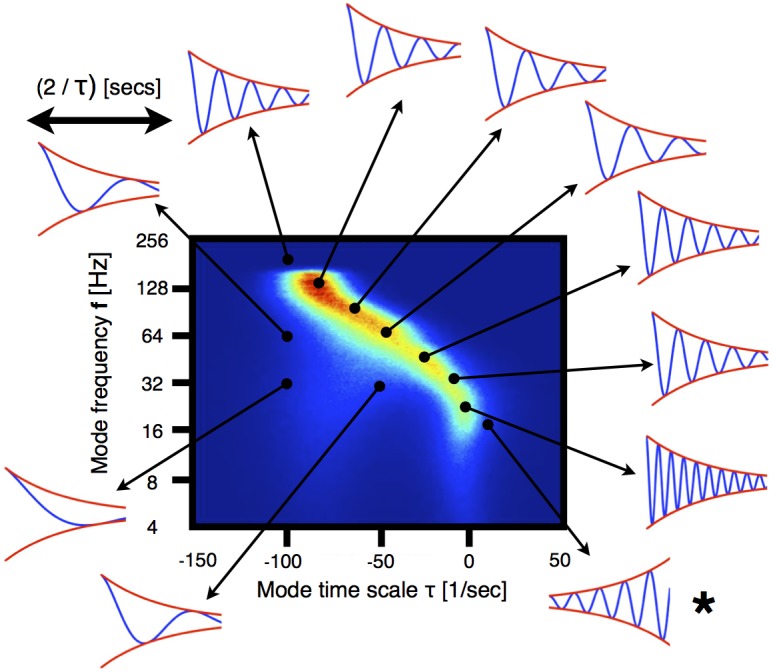
**Qualitative behavior of eigenmodes**. The histogram corresponds to the eigenmodes of VAR-1 processes fitted to ECoG signals. The count in each bin is color-coded and the number of samples is *N* > 10^6^. The frequency axis is in logarithmic scale (base-2). The arrows indicate points in the stability plane for which the qualitative dynamics of the corresponding mode is illustrated. The dynamics of each mode can be expressed as an oscillation of frequency *f* whose amplitude (red curves) is modulated by an exponential decay/growth (blue curve). Each solution is shown for 2/τ s. Note that for the points in the plane with non-zero count, a number of oscillations occur before the mode is damped out. For the case labeled with ⋆, the mode grows exponentially (i.e., it is super-critical).

While Figure 1B suggests an increase in the bulk frequency and decrease in the time constant, this does not fully characterize the way in which anesthetics change the distribution of eigenvalues in the plane spanned by timescale and frequency. Figure 2 shows how we represent the distributions of the eigenvalues of *A*_*j*_. The vertical axes corresponds to frequencies plotted on a logarithmic (base 2) scale. Horizontal axes indicate the modes damping/growth timescale. The sign indicates whether the mode's amplitude is growing (positive) or decaying (negative). Histograms are color coded with blue indicating low occupancy to red indicating high occupancy. Note that the damping time and frequency are not independent and modes with lower frequencies tend to have longer damping times, with slow oscillations found near the critical point (τ ≈ 0). Traces on the margin of the figure illustrate the dynamics for particular pairs of damping time and frequency. Note that the traces are plotted on the timescale commensurate to the damping time rather than on an absolute time scale. The inter-relationship between damping time and frequency assures that most modes located along the most densely populated ridge go through several complete cycles before being damped out, while the modes located to the left of the dominant ridge are damped out earlier and are thus less likely to carry out meaningful computations performed by the brain.

**Figure 3:**
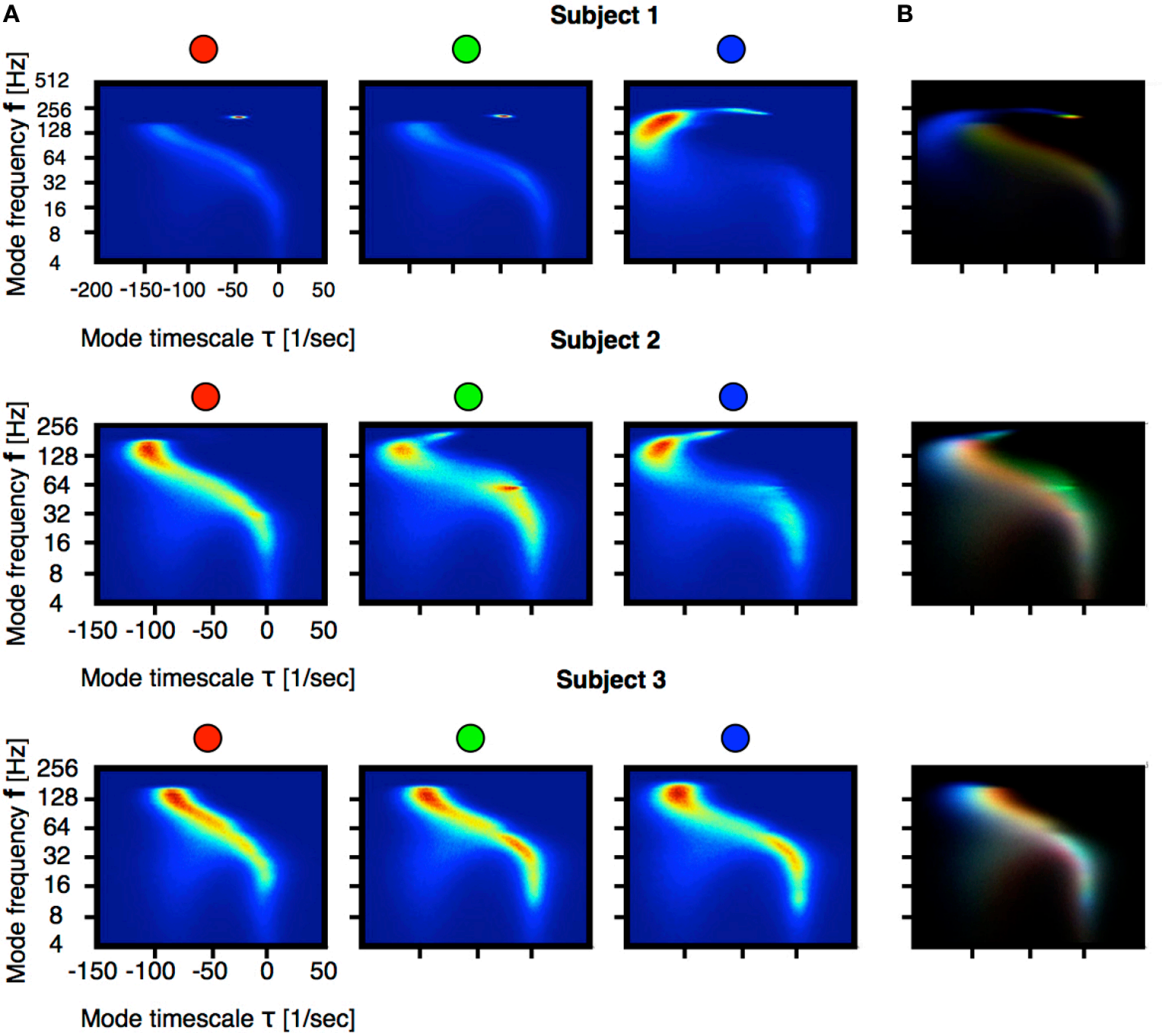
**Statistics of the models stability at each stage indicated in Figure 1**. Each stage is sampled in 100 s intervals and VAR(1) models were fitted every 1 ms. For each stage we computed the frequency and growth \decay timescale of each mode. Figures correspond to 2D histograms of this quantities. The count in each bin is color coded and the number of samples is *N* ≈ 10^6^. The frequency axis is on logarithmic (base 2) scale. **(A)** Distributions of eigenvalues. **(B)** Differences across segments. We normalized each bin in **(A)** histograms by their maximum. The figure is constructed by superposing the three histograms, each coding for a color in RGB space. In this way pure red, pure green and pure blue correspond to eigenvalues that are only present in the first, second and third stage. The rainbow-like pattern indicates a shift of high frequency modes as they become more damped.

**Figure 4:**
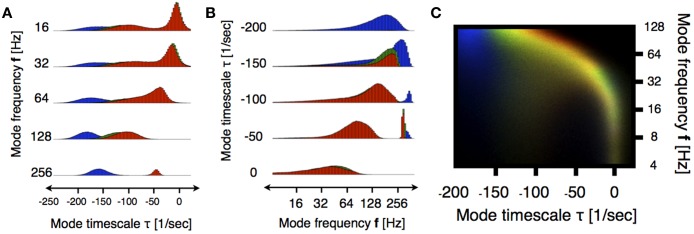
**Detailed view of eigenmodes distributions for subject 1**. Colors indicate the segments indicated in Figure 1A. **(A)** Histogram in Figure 3A is sliced by restricting the analysis to frequency bands of 5 Hz centered around the indicated values. Note that the blue histograms are always to the left of the red ones indicating increased stability as anesthesia is induced. **(B)** Same analysis as **(A)** restricted to slices of 50 $\frac{1}{s}$. Note the emergence of highly damped high frequency oscillations in the anesthetized condition (blue). In both cases we plot count number on the scale (0.46000) **(A)** and (0.120000) for **(B)**. **(C)** Similarly to Figure 3B, we computed the superposition of histograms in a smaller frequency range for better visualization. The histograms were done with logarithmic binning and the frequency axis is logarithmic (base 2). The rainbow indicates a shift toward more damped states. The organization of stability undergoes non-trivial changes in low frequency bands (4–128 Hz).

Figure 3A shows the distribution of eigenvalues in the plane introduced in Figure 2 during three stages of the induction process (100 s segments shown in Figure 1A). In order to better resolve the distributions we performed a moving VAR analysis with *t*_*j* + 1_ − *t*_*j*_ = 1 ms of spacing between adjacent windows. In order to visualize changes in the eigenvalue distributions we normalized the count value of each histogram by its maximum. Then, we used the normalized values in each bin to code for color in RGB space as indicated in the filled circles. Figure 3B, correspond to the superposition of such images. In this way, regions of the stability space that are similarly occupied in the three stages are coded in gray scale [with white corresponding to maximal occupancy (1,1,1)] and pure colors RGB correspond to values that are exclusive to the first, second and third stage respectively. A prominent feature shown by these panels is the shift of high-frequency eigenmodes toward increased stability. While the full worm-like distribution of eigenvalues changes in subtle ways, the left-ward shift in these frequencies is ubiquitous in all subjects. Figures 4A,B correspond to vertical and horizontal “slices” respectively of the histogram shown in Figure 3A for subject 1. Figure 4C shows details of how these distributions change for subject 1. We performed the same comparison as before but choosing a smaller frequency range for computing the histograms. The shift to damped states is more pronounced for modes with frequencies that are greater than 64 Hz.

**Figure 5:**
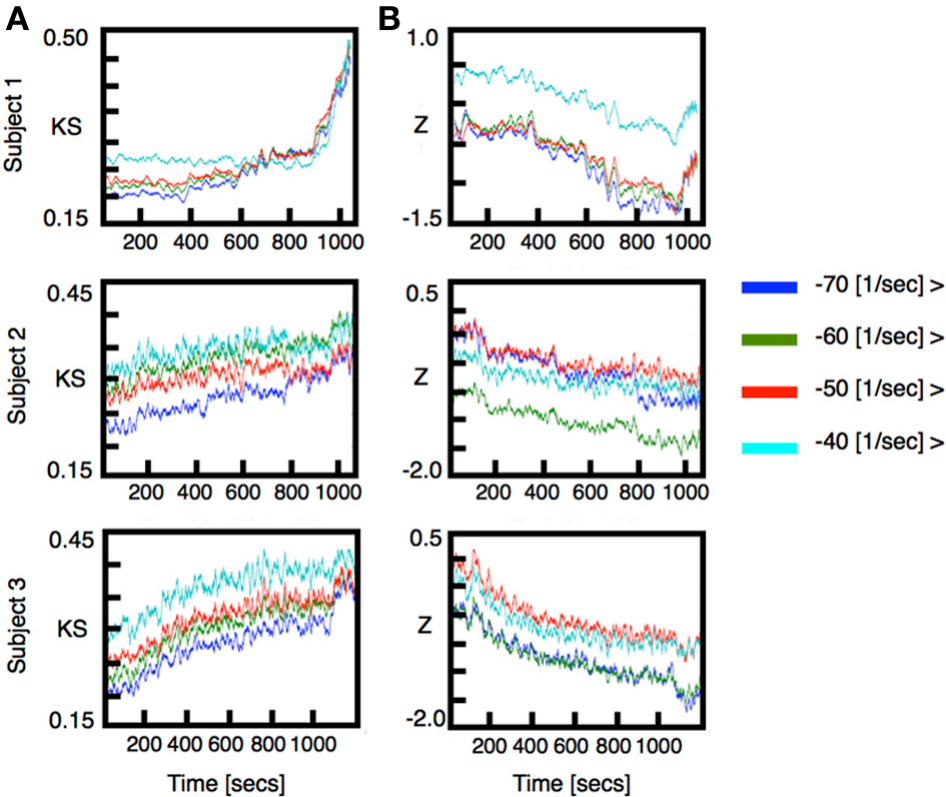
**Stability analysis for truncated distributions of eigenvalues**. We performed the same analysis as in Figure 1A for truncated distributions of eigenvalues; we kept all the eigenvalues with damping constant greater than a threshold. Each color corresponds to different threshold as indicated in the figure labels. **(A)** KS statistics of comparing the truncated distribution of eigenvalues. **(B)** Same as **(A)** using Wilcoxon *z*-value statistics.

Finally, we investigated how the distribution of the most critical modes is affected by induction. This was partially inspired by results previously reported in human ECoG, showing that differences between task and resting conditions can be detected precisely by changes in these populations (Solovey et al., 2012). We show in Figure 5 the result of comparing the distribution of modes truncated to eigenvalues with damping constant above a given threshold close to criticality. For all subjects, the distributions show a *gradual* change in the stability of near-critical modes along the entire span of the induction process Figure 5A. This is somewhat surprising, as the induction process is controlled by *discrete* events of drug increase which notably affect the full eigenmode distribution.

## 4. Conclusions

Dynamical systems theory indicates that systems that are capable of performing computations should have a large number of modes with marginal stability. In such a scenario an arbitrary perturbation will not decay or explode, thus allowing for information integration across the entire system. Previous work suggest that the brain might operate in a dynamically critical regime (Magnasco et al., 2009; Solovey et al., 2012). A simple model exhibiting complex spatio-temporal dynamics was recently proposed, in which statistically critical behavior emerges due to dynamical instabilities. Within this framework we tested the hypothesis that the stability properties of the system change as anesthesia is induced; specifically, we hypothesized that wakefulness is related to dynamical criticality while the anesthetized state corresponds to increased damping of the dynamics. To test this hypothesis we assumed locally linear dynamics estimated in short segments of the recordings using eigenmode decomposition of VAR models.

We found that as the subjects become anesthetized the linear stability of the ECoG recordings show significant changes which are efficiently tracked by non-parametric statistical methods. These markers are remarkably robust to changes in the way data is normalized (choice of filters, amplitude normalization, sampling frequency). Moreover, changes in this quantities were found to be consistent with the subjects behavior as reported by the medical team. This suggests that our indicators could be used to monitor depth of anesthesia.

Our results are also consistent with the criticality hypothesis: we found a prevalence of modes close to criticality across the whole induction procedure. However, as the subjects became anesthetized there were significant changes in the stability properties of the fitted dynamics. These changes were examined closely in selected stages of the procedure and are visualized by the superposed histograms in Figures 3B, 4C. This analysis revealed that changes in the stability exhibit much richer structure than a simple block shift to damping across all frequencies. Yet, we observe a consistent pattern in all three subjects; the eigenvalues of the fitted models shift toward higher frequencies and increased damping. This should be interpreted carefully; it is not necessarily the case that there is an increase of high frequency spectral content of the ECoG signals. Although there ought to be a relationship between a moving spectral analysis and the eigenmodes of a moving VAR analysis, this relationship may be complex.

The increase in the prevalence of eigenmodes characterized by high frequency (high gamma) may be seen as surprising given the well-known observation that the power of high frequency oscillations tends to decrease with some anesthetics including propofol. This result, however, ought to be interpreted carefully. The increase in the number of eigenmodes does not equate to the increase in power. For instance, there could be fractionation of a single correlated pattern of high frequency oscillations in the awake state into multiple mutually independent patterns of high frequency oscillations.

The finding that high frequency modes become more damped as the subject is anesthetized is to some extent reassuring. If we adopt the traditional view that high frequency activity is associated to cognitive processes our results are consistent with an appealing interpretation. The effect of the anesthetic procedure is to damp out high frequency activity while still allowing for low frequency modes to perform a function. Low frequency activity can then presumably be associated to the maintenance tasks which keep the subject alive.

A number of recent reports have been aimed at characterizing criticality as a universal feature in ECoG recordings (He et al., 2010), and as particularly relevant to differentiate wakefulness from sleep (Meisel et al., 2013; Tagliazucchi et al., 2013) (see also Ribeiro et al., 2010 for comparable results with action potential recordings). In this context, our results provide support for a consistent and theoretically founded interpretation of the relationship between criticality and wakefulness. While the theoretical model is not the focus of the present publication, it is interesting to note that it implies a specific and falsifiable prediction: the model achieves self-tuned criticality by means of plastic synaptic adaptation. It follows that blocking synaptic changes should result in a breakdown of criticality; similarly, the model should also be able to explain changes in criticality during the sleep cycle, given the concomitant changes in plasticity patterns (Ribeiro et al., 2007). This will be the subject of future publications, along with further validation of the stabilizing effect of anesthesia in animal models, effects of different anesthetic agents, larger number of subjects, recovery from anesthesia, and application of the methods to EEG recordings.

## 5. Author contributions

Leandro M. Alonso, performed data analysis; Leandro M. Alonso, Alex Proekt, Guillermo A. Cecchi, and Marcelo O. Magnasco wrote the manuscript. Alex Proekt, Guillermo A. Cecchi, Marcelo O. Magnasco designed the experiments; Alex Proekt, Kane O. Pryor, and Theodore H. Schwartz performed the experiments; Leandro M. Alonso and Alex Proekt contributed equally to this work.

### Conflict of interest statement

The Guest Associate Editor A. Ravishankar Rao declares that, despite being affiliated to the same institution as the author Guillermo A. Cecchi, the review process was handled objectively and no conflict of interest exists. The authors declare that the research was conducted in the absence of any commercial or financial relationships that could be construed as a potential conflict of interest.
